# Photogenerated charges and surface potential variations investigated on single Si nanorods by electrostatic force microscopy combined with laser irradiation

**DOI:** 10.1186/1556-276X-9-245

**Published:** 2014-05-20

**Authors:** Shan Wu, Zilong Wu, Dongdong Lin, Zhenyang Zhong, Zuimin Jiang, Xinju Yang

**Affiliations:** 1 State Key Laboratory of Surface Physics, Fudan University, Shanghai 200433, China

**Keywords:** Si nanorods, EFM, Photogenerated charging, Surface potential

## Abstract

Photogenerated charging properties of single Si nanorods (Si NRs) are investigated by electrostatic force microscopy (EFM) combined with laser irradiation. Under laser irradiation, Si NRs are positively charged. The amount of the charges trapped in single NRs as well as the contact potential difference between the tip and NRs' surface is achieved from an analytical fitting of the phase shift - voltage curve. Both of them significantly vary with the laser intensity and the NR's size and construction. The photogenerated charging and decharging rates are obtained at a timescale of seconds or slower, indicating that the Si NRs are promising candidates in photovoltaic applications.

## Background

One-dimensional silicon nanostructures, such as Si nanowires (NWs), nanorods (NRs), or nanopillar (NPs) have gained particular interests due to their special properties and potential applications in electronic and optoelectronic devices [1-4]. Theoretical and experimental studies have reported that when arranged in a highly ordered fashion, Si NRs or NWs can improve light absorption and charge collection, making it possible to achieve high efficiency in solar cells [5-8]. Therefore, periodic Si NRs (or NWs) arrays have attracted considerable attentions in the fields of solar cells. However, despite the huge efforts to control and understand the growth mechanisms underlying the formation of these nanostructures [9,10], some fundamental properties and inside mechanisms are still not well understood.

To reveal their properties, the investigation on single NRs is preferred. Recently conductive scanning probe microscopy techniques have been attempted to investigate the electrical properties of single NWs/NRs. Among them, electrostatic force microscopy (EFM) can provide direct information of trapped carriers in single nanostructures and has been applied to investigate the charge trapping in single nanostructures, such as carbon nanotubes [11], pentacene monolayer islands [12], CdSe quantum dots (QDs) [13,14], and etc. More recently, photoionization of QDs [15,16] and photo-induced charging of photovoltaic films [17-19] have been studied by EFM combined with laser irradiation. But the photogenerated charging effects have not been concerned on Si NRs or NWs yet. In this letter, EFM measurements combined with laser irradiation are applied to investigate the photogenerated charging properties on single vertically aligned Si NRs in periodic arrays.

## Methods

**Figure 1:**
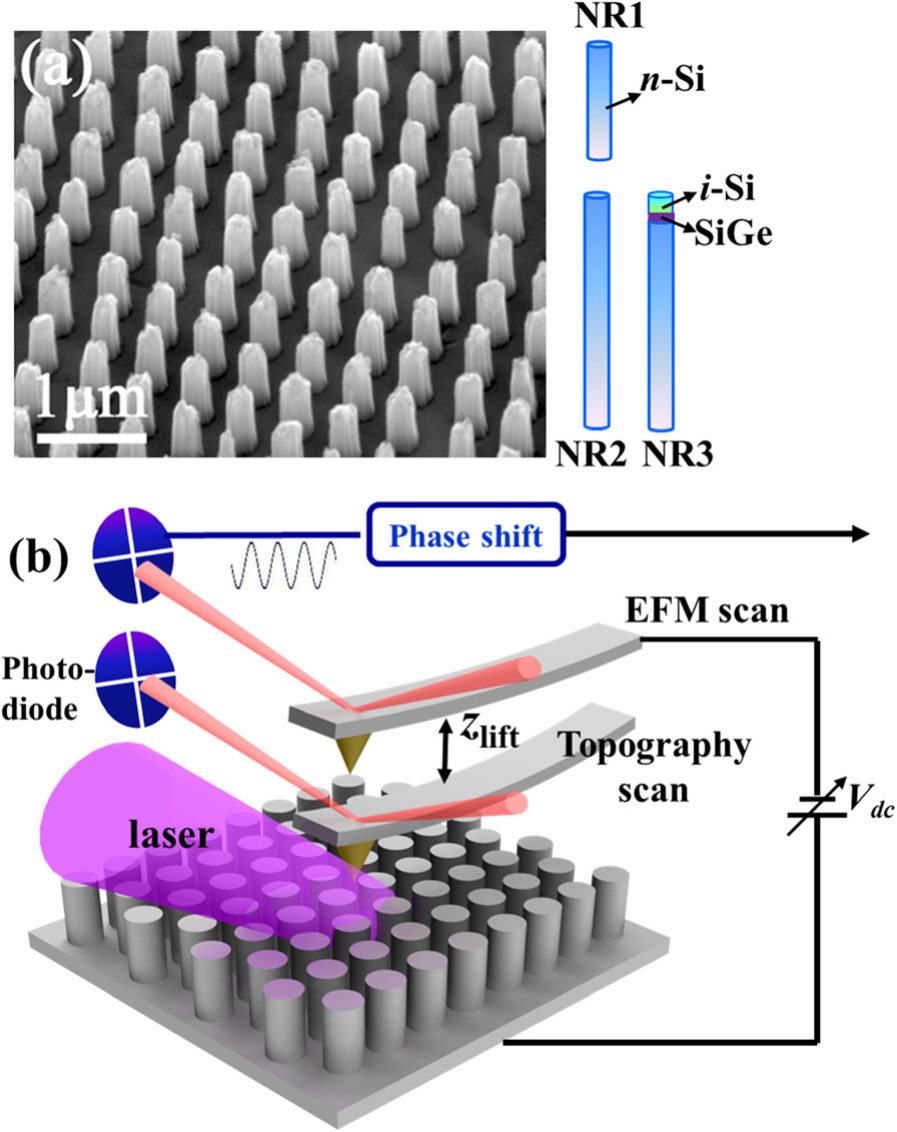
**Constructions of NRs and schematic diagram of EFM measurements. (a)** SEM image of NR2, together with the constructions of NR1, NR2, and NR3. **(b)** Schematic diagram of EFM measurements on single Si NRs combined with a 405-nm laser irradiation.

Periodic arrays of Si NRs are fabricated by nanosphere lithography and metal-assisted chemical etching. Three samples (labeled as NR1, NR2, NR3) which contain periodic NR arrays with the same diameter of about 300 nm and different length or constructions are prepared. NR1 and NR2 are n-type Si (approximately 1,000 Ω cm) NRs with the length of about 0.5 and 1.0 μm, respectively, while NR3 is Si/SiGe/Si hetero-structural NRs with the length of 1.0 μm, which is fabricated with the same n-type Si substrate but covered with a 5-nm Si_0.55_Ge_0.45_ quantum well and a 100-nm intrinsic Si capping layer [20]. The constructions of three types of NRs are given in Figure 1a, together with the scanning electron microscopy (SEM) image of NR2. The SEM images of NR1 and NR3 are similar to that of NR2, except the length of NR1 is smaller than the other two. Figure 1b gives an experimental schematic diagram of EFM measurements on single Si NRs combined with laser irradiation. The phase shift vs. voltage (*ΔΦ − V*_EFM_) curves are measured at a lift height on single NRs with SCM-PIT tips. Laser (405 nm) with adjustable power intensity is focused onto the substrate through a 400-μm fiber, with a spot of about 1 mm^2^ at the area beneath the AFM tip. All measurements are operated in a nitrogen flow gas for a stable measurement.

## Results and discussions

**Figure 2:**
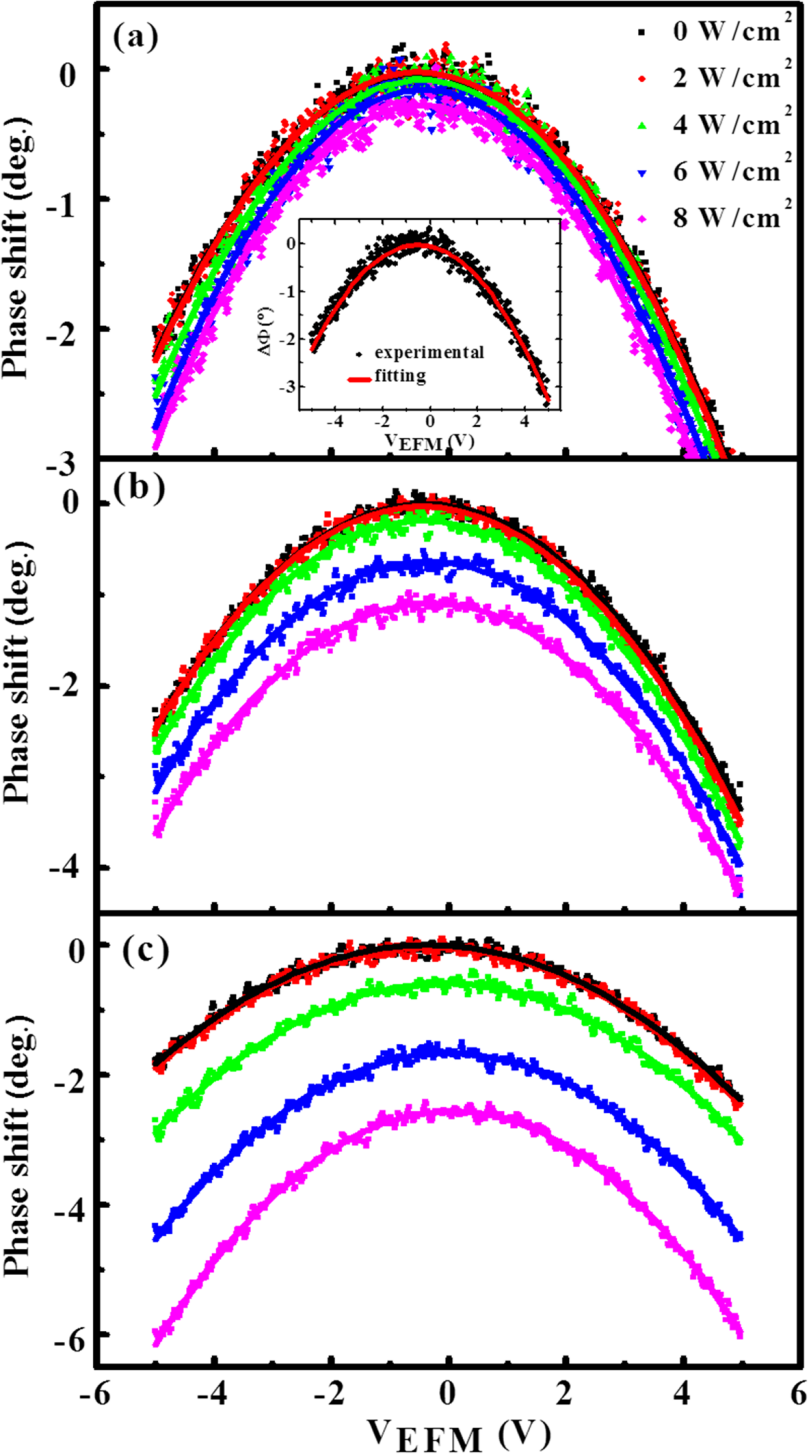
***ΔΦ − V***_**EFM **_**curves measured at different laser intensities for NR1 (a), NR2 (b), and NR3 (c).** The experimental data are plotted with scattered dots, and the fitting results are given with lines. A fitting example of NR1 without laser is presented in the inset of (a).

The *ΔΦ − V*_EFM_ curves measured at a lift height of 140 nm on three samples under different laser intensities are shown in Figure 2 as the scattered dots. It can be seen that the curves shift to the negative direction with the laser intensity, and the shift varies with the type of the NRs. In previous literatures, the relation between phase shift and electrostatic force has been established, where the tip-sample system is simply treated as plane capacitor [21-23]. When a bias is applied between the tip and the sample, the capacitive electrostatic force gradient would cause a phase shift. If there are charges trapped in the sample, additional phase shift induced by the coulombic force is generated. Therefore, at the lifted pass where the Van der Waals force can be ignored, the force on the tip can be written as [11,24,25]:

(1) $$\begin{array}{ll} F_{\mathrm{Electrostatic}} & =F_{\mathrm{Capacitive}}+F_{\mathrm{Coulombic}}=\frac{1}{2}\frac{\partial C}{\partial z}{\left(V_{\mathrm{EFM}}-V_{\mathrm{CPD}}\right)}^{2}\\ & -\frac{CQ_{s}}{4\pi \epsilon_{0}z^{2}}\left(V_{\mathrm{EFM}}-V_{\mathrm{CPD}}\right)-\frac{Q_{s}^{2}}{4\pi \epsilon_{0}z^{2}} \end{array}$$

Where *C*, *V*_EFM_, and *V*_CPD_ are the capacitance, applied DC voltage, and contact potential difference (CPD) between the tip and sample, respectively. *Q*_*s*_ is the amount of charges trapped in the beneath NR, and *z* is the distance between the trapped charges in NR and image charges in tip. The phase shift detected by EFM is proportional to the gradient of the force, which is as follows:

(2) $$\begin{array}{ll} \Delta \Phi & =-\frac{Q}{k}\frac{\partial F}{\partial z}=-\frac{Q}{k}\left[\frac{1}{2}\frac{\partial^{2}C}{\partial z^{2}}{\left(V_{\mathrm{EFM}}-V_{\mathrm{CPD}}\right)}^{2}\right]\\ & +\frac{Q_{s}}{2\pi \epsilon_{0}z^{2}}\left(\frac{C}{z}-\frac{1}{2}\frac{\partial C}{\partial z}\right)\left(V_{\mathrm{EFM}}-V_{\mathrm{CPD}}\right)+\frac{Q_{s}^{2}}{2\pi \epsilon_{0}z^{3}} \end{array}$$

where *Q* is the quality factor and *k* is the spring constant of the probe.

From Equation 2, it can be seen, without charges trapped in Si NRs, that the EFM phase shift should be equal to zero at *V*_EFM_ = *V*_CPD_. In other words, the minimum point of the *ΔΦ − V*_EFM_ curve should be located at zero. In Figure 2, the *ΔΦ − V*_EFM_ curves of three types of NRs did present their minimum points at almost zero without laser irradiation, indicating that the trapped charges can be neglected in that case. However, with laser irradiation, all *ΔΦ − V*_EFM_ curves of the three samples gradually decline to negative sides, suggesting charges are generated by laser irradiation and trapped in Si NRs. From Figure 2, it can also be observed that the decline of phase shift increases with the laser intensity, and the range of decline is significant different for the three types of NRs. To achieve the amount of the trapped charges, curve fittings are made by using Equation 2. Let: $A=-\frac{Q}{2k}\frac{\partial^{2}C}{\partial z^{2}}$, $B=-\frac{Q}{k}\frac{Q_{s}}{2\pi \epsilon_{0}z^{2}}\left(\frac{C}{z}-\frac{1}{2}\frac{\partial C}{\partial z}\right)$, and $C=-\frac{Q}{k}\frac{Q_{s}^{2}}{2\pi \epsilon_{0}z^{3}}$, Equation 2 is simplified to:

(3) $$\Delta \Phi =A{\left(V_{\mathrm{EFM}}-V_{\mathrm{CPD}}\right)}^{2}+B\left(V_{\mathrm{EFM}}-V_{\mathrm{CPD}}\right)+C$$

**Figure 3:**
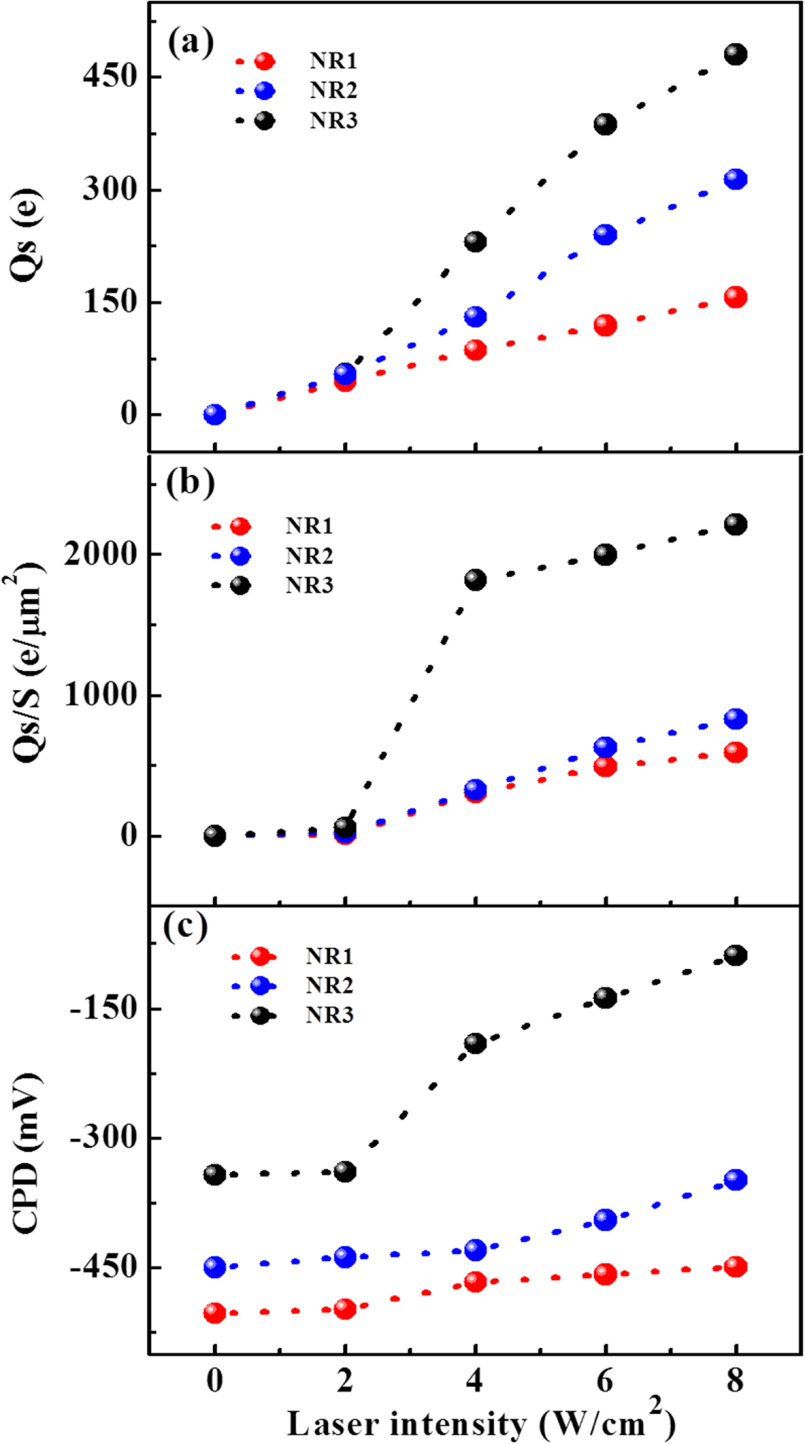
**The trapped charges Q**_**s **_**(a), charge density (b) and CPD values (c).** Of the three samples as a function of laser intensity.

By using Equation 3 and treating *A*, *B*, *C*, and *V*_CPD_ as fitting parameters, the *ΔΦ − V*_EFM_ curves of the three samples under different laser intensities can be well fitted, shown as the lines in Figure 2. A fitting example of NR1 without laser irradiation is given in the inset of Figure 2a, and the results of the fitting parameters for NR1, NR2, and NR3 are given in Tables 1, 2, and 3, respectively. From the fitting parameter *C*, the trapped charges *Q*_*s*_ can be simulated by using *Q* = 186 and *k* = 2.8 N/m for PIT tip [13,14] and approximating *z* as the lift height, as plotted in Figure 3a as a function of laser intensity. Under 2 W/cm^2^ laser irradiation, the amount of charges trapped in single NR1, NR2, and NR3 are 32, 54, and 55 e, respectively. It increases quickly when the laser intensity increases above 4 W/cm^2^, particularly for NR3. It is obtained that under 8 W/cm^2^ laser irradiation, the trapped charges in single NR1, NR2, and NR3 increase to 149, 314, and 480 e, respectively. Here, it should be noted that these values are very imprecise, as the exact distance between the trapped charges in NR and image charges in tip cannot be obtained in our experiments and it is roughly treated as the lift height, i.e., 140 nm. Therefore, the real trapped charges should be larger than that the preceding values due to the larger value of real *z*. Meanwhile, from the preceding descriptions of B and C, the relation between B and C can be written as: $C=-\frac{k}{Q}\frac{2\pi \epsilon_{0}z}{{\left(C/z-\left(1/2\right)\left(\partial C/\partial z\right)\right)}^{2}}B^{2}$. From the fitting results of B and C as listed in Tables 1, 2, and 3, a well quadratic fitting of C with B can be achieved (not shown here), ensuring that the above analytical fitting model is suitable for our results and the phase shift under laser irradiation is corresponding to the charging effect.

**Table 1:** **Fitting results obtained by fitting *ΔΦ − V***_**EFM **_**curves of NR1 with Equation** 3

**Laser intensity (W/cm**^**2**^**)**	**A**	**B**	**CPD (V)**	**C**	**Q**_**s **_**(e)**	***Q***_***s***_**/*S *** **(e/μm**^**2**^**)**
0	−0.1070	0.0000	−0.503	0.0000	0	0
2	−0.1100	0.0002	−0.498	−0.0114	32	13
4	−0.1172	0.0051	−0.467	−0.0822	86	307
6	−0.1240	0.0086	−0.458	−0.1378	111	489
8	−0.1288	0.0108	−0.449	−0.2480	149	591

**Table 2:** **Fitting results obtained by fitting *ΔΦ − V***_**EFM **_**curves of NR2 with Equation** 3

**Laser intensity (W/cm**^**2**^**)**	**A**	**B**	**CPD (V)**	**C**	**Q**_**s **_**(e)**	***Q***_***s***_**/*S *** **(e/μm**^**2**^**)**
0	−0.1162	0.0000	−0.450	0.0000	0	0
2	−0.1174	0.0004	−0.438	−0.0319	54	24
4	−0.1210	0.0056	−0.433	−0.1835	129	325
6	−0.1169	0.0104	−0.395	−0.6365	239	627
8	−0.1138	0.0134	−0.349	−1.0935	314	830

**Table 3:** **Fitting results obtained by fitting *ΔΦ − V***_**EFM **_**curves of NR3 with Equation** 3

**Laser intensity (W/cm**^**2**^**)**	**A**	**B**	**CPD (V)**	**C**	**Q**_**s **_**(e)**	***Q***_***s***_**/*S *** **(e/μm**^**2**^**)**
0	−0.0840	0.0000	−0.343	0.0000	0	0
2	−0.0853	0.0007	−0.339	−0.0335	55	58
4	−0.0947	0.0244	−0.191	−0.5880	230	1817
6	−0.1148	0.0325	−0.138	−1.6667	387	1996
8	−0.1403	0.0440	−0.089	−2.5633	480	2212

Furthermore, the trapped charge density can be also estimated from the ratio of the fitting parameters A and B by using a recently proposed analytical mode dealing with nanoparticles [21]. When considering the nanoparticle as a thin dielectric layer of height *h* and dielectric constant *ϵ* and approximating that *h*/*ϵ* < < *z*, the parameters A and B could be written as:

(4) $$A=-\frac{Q}{2k}\frac{3\epsilon_{0}\mathrm{Sh}}{z^{4}},\;B=\frac{Q}{2k}\frac{Q_{s}h}{\epsilon z^{3}}$$

From Equation 4, the trapped charges *Q*_*s*_ can be also derived via B if taking the *h* as the height of NRs. But the obtained values are smaller than those derived from C for all the three samples, especially for NR2 and NR3. It may be due to the charges that are only trapped in a top part of the NR, and the exact value of *h* is smaller than the NR's height. But the real height of *h* could not obtained in our experiment, thus instead the ratio B/A was applied to simulate the charge density which ignores the influence of *h*. After taking the nanostructure and tip shapes into account, one can obtain [12,21].

(5) $$\frac{B}{A}=-\frac{g}{\alpha }\frac{\left(Q_{s}/S\right)z}{\epsilon_{0}\epsilon_{r}}$$

The tip shape factor, α, is about 1.5 for a standard conical tip [12,21]. The NRs' shape factor, *g*, is about 1 if we approximate the NRs as cylindrical nanoparticles [21]. *Q*_*s*_*/S* is the trapped charge density to be derived, and *ϵ*_*r*_ is the dielectric constant of Si. Thus, the charge densities can be obtained by using Equation 5, which are listed in Tables 1, 2, and 3 and also plotted as a function of laser intensity in Figure 3b. The results show a similar tendency of increase with the laser intensity as the trapped charges as given in Figure 3a, except the increase of tapped charge density in NR3 is much larger than that of the trapped charges, which may be due to more localization of charges in NR3. Again, the obtained values are not accurate due to the uncertainty of *z*.

In addition, from the description of B in Equation 4, the polarity of *Q*_s_ can be obtained from the sign of B. From the fitting results, it is obtained that B increases from zero to positive values with the laser intensity for all the three samples, indicating that positive charges are trapped in the three types of NRs under laser irradiation. The increase of trapped charges is relatively small for NR1, which should be again due to its low absorbance of light. The reason why the NR3 contains more trapped charges than NR2 is most probably due to the existence of the GeSi quantum well, which can act as additional trappers of holes.

On the other hand, the values of *V*_CPD_ can also be obtained from the fitting results, and the change of *V*_CPD_ with laser intensity is presented in Figure 3c. It can be observed that, under 2 W/cm^2^ laser irradiation, the *V*_CPD_ values change slightly for all the three samples, but they increase obviously when the laser intensity increase up to 4 W/cm^2^ and above. Also, the increase magnitude is different for the three types of NRs. The increase of *V*_CPD_ with laser intensity is most significant for NR3, similar to the increase of trapped charges. Similar surface potential variation by photogenerated charges has been obtained by Kelvin potential force microscopy (KPFM) [26,27]; it was declared that the positive (negative) shift in surface potential with laser corresponds to an increase in hole (electron) density. Thus, the positive shift in *V*_CPD_ with laser intensity in our experiments can also be attributed to the increase of trapped hole density, which is consistent with the above results of charge density. As *V*_CPD_ equals to (*ϕ*_tip_ − *ϕ*_sample_) / e, the results declare that the work function of Si NR decrease upon laser irradiation should be due to the photogenerated holes trapped in NRs.

The reason why positive charging measured on n-type Si NRs is not very clear, and further studies are required to get a clear mechanism. The possible mechanism may be suggested to the tunneling of photogenerated electrons to the substrate and trapping the holes in the NRs. In previous studies on the photoionization of an individual CdSe nanocrystals [16,28], it was found that a significant fraction of nanocrystals was positively charged and it was attributed to the tunneling of the excited electrons into the substrate. They assumed that the hole tends to be localized in the nanocrystal, while the electron is much more delocalized, with a nonnegligible fraction of the electron density outside the nanocrystal. Another possibility arises from that the holes can be captured at Si-Si bonds according to the reaction ≡ Si-Si ≡ + *h →* ≡Si^+^ + · Si≡, as reported in reference [29]. By adopting the above viewpoint, it can be suggested that when Si NRs are irradiated, free charges are photogenerated after dissociation of the excitons. Due to the tunneling of photoelectrons and/or capture of holes, the Si NRs would be positively charged.

**Figure 4:**
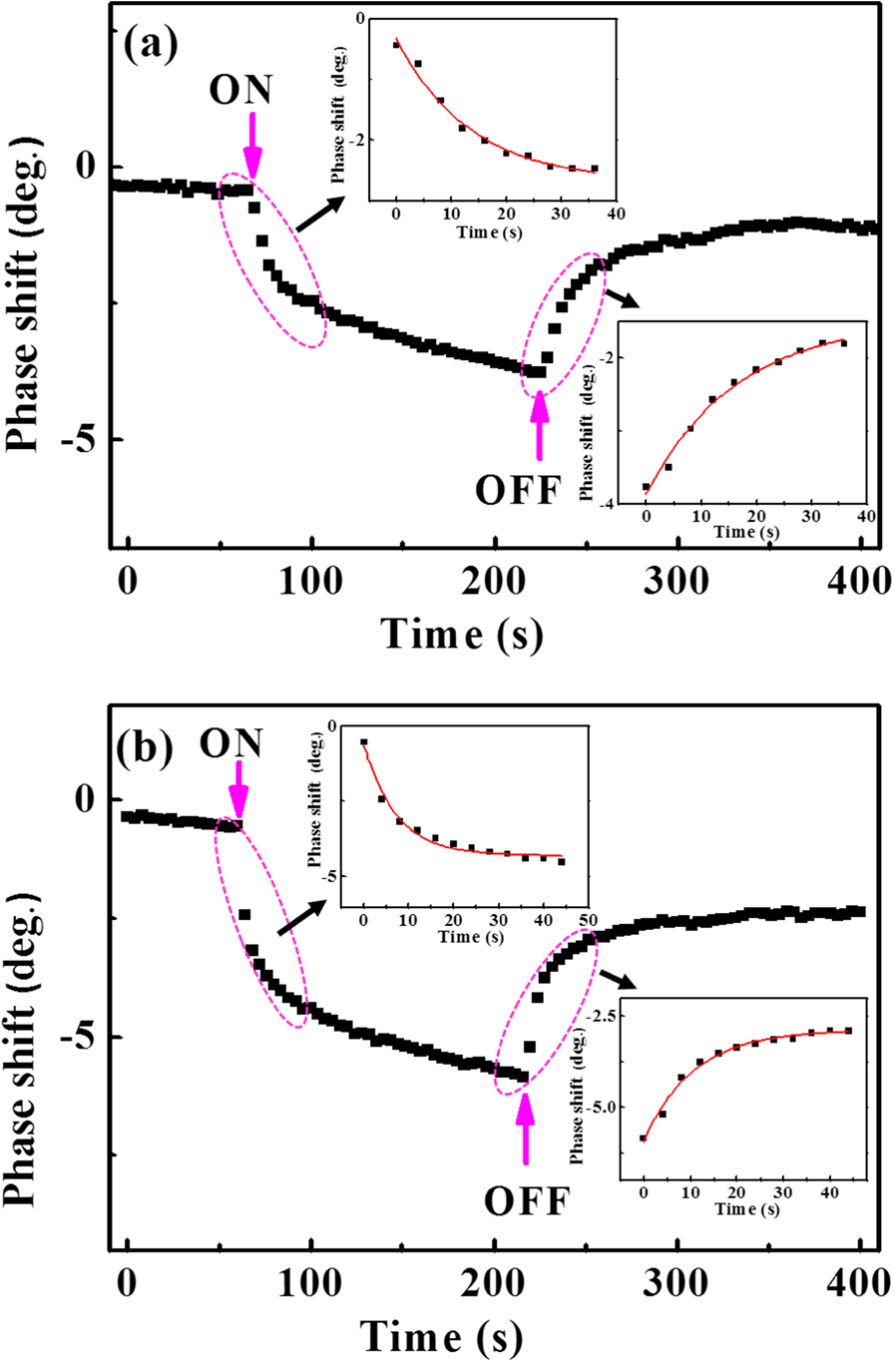
**Time evolutions of EFM phase shift.** Of NR2 **(a)** and NR3 **(b)** obtained at a sample bias of 2 V when the laser is ON and OFF. The exponential decay and growth fittings of the data when the laser is ON and OFF are given in the insets of the figure.

To see the dynamics of charging and decharging, the time evolution of the EFM phase shift with the laser ON and OFF is present in Figure 4a,b for NR2 and NR3, respectively. As the change of phase shift with laser irradiation is too small for NR1, it is not given here. When the laser is turned on, the EFM phase shifts of both NR2 and NR3 moves to the more negative values, and the signal follows a monotonic decay to a new equilibrium value, corresponding to the charge generation and trapping process. The experimental curves can be fitted with single exponential decay, as shown in the left insets in Figure 4, giving a time constant of 7.6 and 13.6 s for NR2 and NR3, respectively. In addition, the time evolution of EFM phase shift after the laser is turned off is also recorded. Upon the removal of the laser light, the separated hole and electron recombine to restore the original phase shift [30]. As shown in the insets of Figure 4, they can be well fitted by single exponential growth, giving a time constant of 10.6 and 16.6 s for NR2 and NR3, respectively. The results indicate that both the charging and decharging rates in Si NRs are very slow, which are at the timescale of seconds. So, periodic Si NRs should have promise application potentials in photovoltaic devices. The time constants of charging and decharging are a little larger for NR3 than NR2, which may be due to the additional charging and decharging process of the quantum well in NR3, suggesting NR3 are especially better for applications.

**Figure 5:**
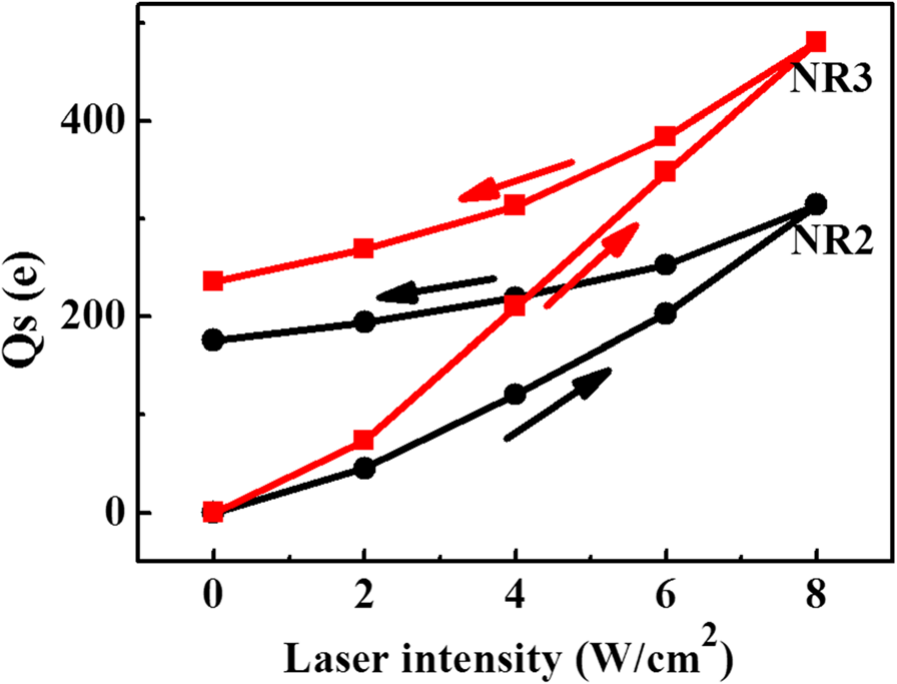
Hysteresis effect of photogenerated charges in NR2 and NR3.

In Figure 4, it can also be observed that for both NR2 and NR3, the stabilized phase shift after the laser turns off is still a little smaller than that before the laser turns on, even after about 200 s. It indicates that another much slower decharging phenomenon should be involved. Thus, the hysteresis effects of the photogenerated charging as a function of laser intensity are measured on both NR2 and NR3, as shown in Figure 5. The laser intensity increases from 0 to 8 W/cm^2^ and subsequently decreases to 0, and at each point, the measurement is taken after about 2 min stabilization. An obvious hysteresis effect as a function laser intensity is observed for both NR2 and NR3, and the amount of stored charges in the backward loop is larger than that in the forward loop, suggesting that this part of charges decays with a slower time than which needed for each measurement. These charges are found to be detrapped after about half an hour. Similar charging hysteresis effect was observed on Si nanoparticles covered with oxide layer by direct charge injection [31], and it was interpreted that charges were stored in the oxide layer of the nanoparticles. As in our case, the NRs are also covered with the native oxide layer; it is also possible that a part of charges are trapped in the oxide layer or interface states which decays slower than the time for each measurement, resulting in the hysteresis in trapped charges. Since this type of charges trapped in NR3 is larger than that in NR2, this difference could be attributed to the existence of GeSi quantum well which increases the interface states.

## Conclusions

In conclusion, the photogenerated charging and trapping phenomena are directly measured on single Si NRs without the deposition of electrodes by the means of EFM combined with laser irradiation. The amounts of photogenerated charges trapped in single NRs and the CPD values are obtained from the analytical fitting of *ΔΦ − V*_EFM_ curves. The quantities of charges and CPD values are found to increase with the laser intensity and vary with the type of NRs. Though the exact mechanism for explaining the photogenerated effects of single Si NRs is not variable at present, it is clear that photoexcitation can lead to obvious charges trapped in Si NRs and hence reduce the work function of NRs. Therefore, EFM can provide an effective way to gain direct information on the trapped charges and surface potential of single nanostructures by combining with laser irradiation, which should be important for both basic understanding and potential applications of nanostructures in optoelectronics and photovoltaics.

## Competing interests

The authors declare that they have no competing interests.

## Authors' contributions

SW carried out the experiments. ZLW prepared the samples. SW and XJY interpreted the results and wrote the manuscript. DDL participated in manuscript preparation. ZYZ and ZMJ helped in interpretation and discussions. All authors read and approved the final manuscript.
